# Fiber optic bronchoscopy in patients with acute hypoxemic respiratory failure requiring noninvasive ventilation - a feasibility study

**DOI:** 10.1186/cc10328

**Published:** 2011-07-27

**Authors:** Hans Jörg Baumann, Hans Klose, Marcel Simon, Tarik Ghadban, Stephan A Braune, Jan K Hennigs, Stefan Kluge

**Affiliations:** 1 Department of Intensive Care Medicine, University Medical Center Hamburg-Eppendorf, Martinistr. 52, Hamburg, 20246, Germany; 2 Department of Respiratory Medicine, University Medical Center Hamburg-Eppendorf, Martinistr. 52, Hamburg, 20246, Germany

## Abstract

**Introduction:**

Noninvasive ventilation (NIV) is a standard procedure in selected patients with acute respiratory failure. Previous studies have used noninvasive ventilation to ensure adequate gas exchange during fiberoptic bronchoscopy in spontaneously breathing hypoxemic patients, thus avoiding endotracheal intubation. However, it is unknown whether bronchoscopy can be performed safely in patients with acute hypoxemic respiratory failure already in need of NIV prior to the decision for bronchoscopy.

**Methods:**

We prospectively investigated 40 consecutive, critically ill, adult patients with acute hypoxemic respiratory failure (14 women, 26 men, age 61 ± 15 years, partial pressure for oxygen/fraction of inspired oxygen (PaO_2_/FiO_2_) < 300 under noninvasive ventilation, Simplified Acute Physiology scores (SAPS II) 47 ± 9.9 points). All patients required noninvasive ventilation prior to the decision to perform bronchoscopy (median 10.5 h; range 2.2 to 114). Blood gases, heart rate, blood pressure and ventilation were monitored before, during and up to 120 minutes after bronchoscopy.

**Results:**

Bronchoscopy could be completed in all patients without subsequent complications. Oxygen saturation fell to < 90% in two patients (5%), and the lowest value during the procedure was 84%. The mean PaO_2_/FiO_2 _ratio improved from 176 ± 54 at baseline to 240 ± 130 (*P *< 0.001) at the end of bronchoscopy and 210 ± 79 after 120 minutes. The transient mean partial pressure of carbon dioxide in the arterial blood (PaCO_2_) increase was 9.4 ± 8.1 mm Hg. Four patients (10%) required endotracheal intubation during the first eight hours after the procedure. Bronchoalveolar lavage yielded diagnostic information in 26 of 38 (68%) patients.

**Conclusions:**

In critically ill patients with acute hypoxemic respiratory failure requiring noninvasive ventilation, bronchoscopy can be performed with an acceptable risk. Since these patients *per se *have a high likelihood of subsequent endotracheal intubation due to failure of NIV, bronchoscopy should only be performed by experienced clinicians.

## Introduction

Noninvasive ventilation (NIV) constitutes a cornerstone in the treatment of acute respiratory failure of various etiologies [1-3]. Some patients treated for acute respiratory failure in the intensive care unit will require diagnostic or therapeutic bronchoscopy. While bronchoscopy is generally considered a safe intervention [4], it is well known that bronchoscopy and especially bronchoalveolar lavage (BAL) may induce significant hypoxemia [5]. Therefore, severe hypoxemia represents a contraindication for bronchoscopy unless it can be reversed by therapeutic interventions. Especially in hypoxemic patients the known benefits of the procedure have to be weighed against possible risks [6].

Small case series and few randomized controlled studies have suggested that NIV can prevent respiratory deterioration in spontaneously breathing hypoxemic patients undergoing bronchoscopy [7-13]. In these studies noninvasive ventilatory support was purely initiated to facilitate bronchoscopy and the study population did not require NIV for hypoxemia at that point in time. However, it is unknown whether bronchoscopy can be performed with acceptable risk in patients with acute hypoxemic respiratory failure already in need of NIV prior to the decision for bronchoscopy. These patients represent a population with substantially more respiratory compromise than those from the previous studies mentioned above [7-13].

We, therefore, conducted a prospective study to assess the feasibility of fiberoptic bronchoscopy in critically ill patients already requiring NIV due to acute hypoxemic respiratory failure prior to the bronchoscopy procedure.

## Material and methods

### Study design

The study was an observational, prospective cohort study. It was approved by the ethics committee of the chamber of physicians in Hamburg, Germany and was performed in accordance with the ethical standards laid down in the Declaration of Helsinki. Patients admitted between November 2007 and January 2010 to the Department of Intensive Care Medicine, University Medical Center Hamburg-Eppendorf were eligible for enrolment into the study. All participants or their legal representatives gave written informed consent.

### Study subjects

The patient population included consecutive medical, neurological and surgical intensive care unit (ICU) patients. Inclusion criteria were: 1) acute hypoxemic respiratory insufficiency with consecutive hypoxemia, defined as PaO_2_/FiO_2 _< 300 under noninvasive ventilation, 2) requiring NIV prior to bronchoscopy, 3) requiring fiberoptic bronchoscopy for diagnostic or therapeutic reasons, 4) age ≥18 years, and 5) informed consent. Patients in whom NIV was instituted purely to facilitate bronchoscopy were excluded. The decision both to initiate NIV and to perform a bronchoscopy was made by the attending intensivist on clinical grounds and in accordance with published guidelines [14]. Simplified acute physiology scores (SAPS II) were calculated using standard criteria [15]. Immunosuppression was defined as absolute neutrophil count < 1,000/mL, immunosuppressive medication, organ transplantation, high dose chemotherapy during the past 60 days, or acquired immunodeficiency syndrome. Standard microbiological diagnostic measures prior to bronchoscopy included blood cultures and urinary antigen assay for *Legionella*. While sputum cultures were rarely used, additional serological studies were ordered in immunosuppressed patients as indicated.

### Noninvasive ventilation

According to inclusion criteria, all patients were on NIV prior to and throughout the bronchoscopy procedure. ICU ventilators were used with the NIV option activated (Evita 4, Dräger, Germany). The ventilator mode was set to pressure support (CPAP/ASB) or pressure controlled mode (BIPAP). Inspiratory pressure was set in the range from 10 to 25 cm H_2_O and positive end-expiratory pressure (PEEP) from 5 to 10 cm H_2_O to achieve optimal ventilation and oxygenation. Ventilator settings were adjusted by the attending intensivists. Simple full-face masks (B+P, Neunkirchen-Seelscheid, Germany) were used as interfaces secured to the patient's face with elastic straps. The time from initiation of NIV to the bronchoscopy procedure was recorded. Following bronchoscopy all patients remained on noninvasive ventilatory support for at least two hours.

### Bronchoscopy procedure

**Figure 1:**
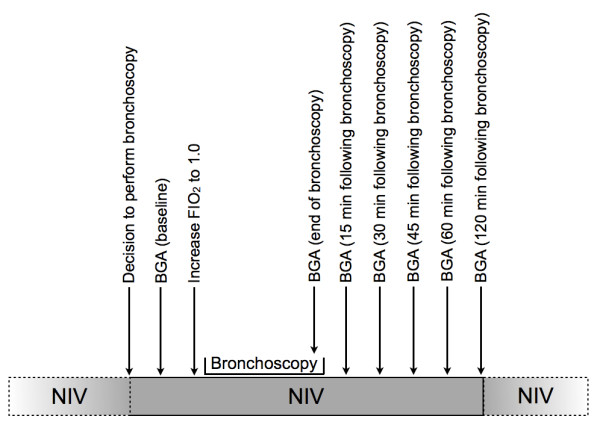
**Design of the study**. NIV, noninvasive ventilation; FiO_2_, fraction of inspired oxygen.

**Figure 2:**
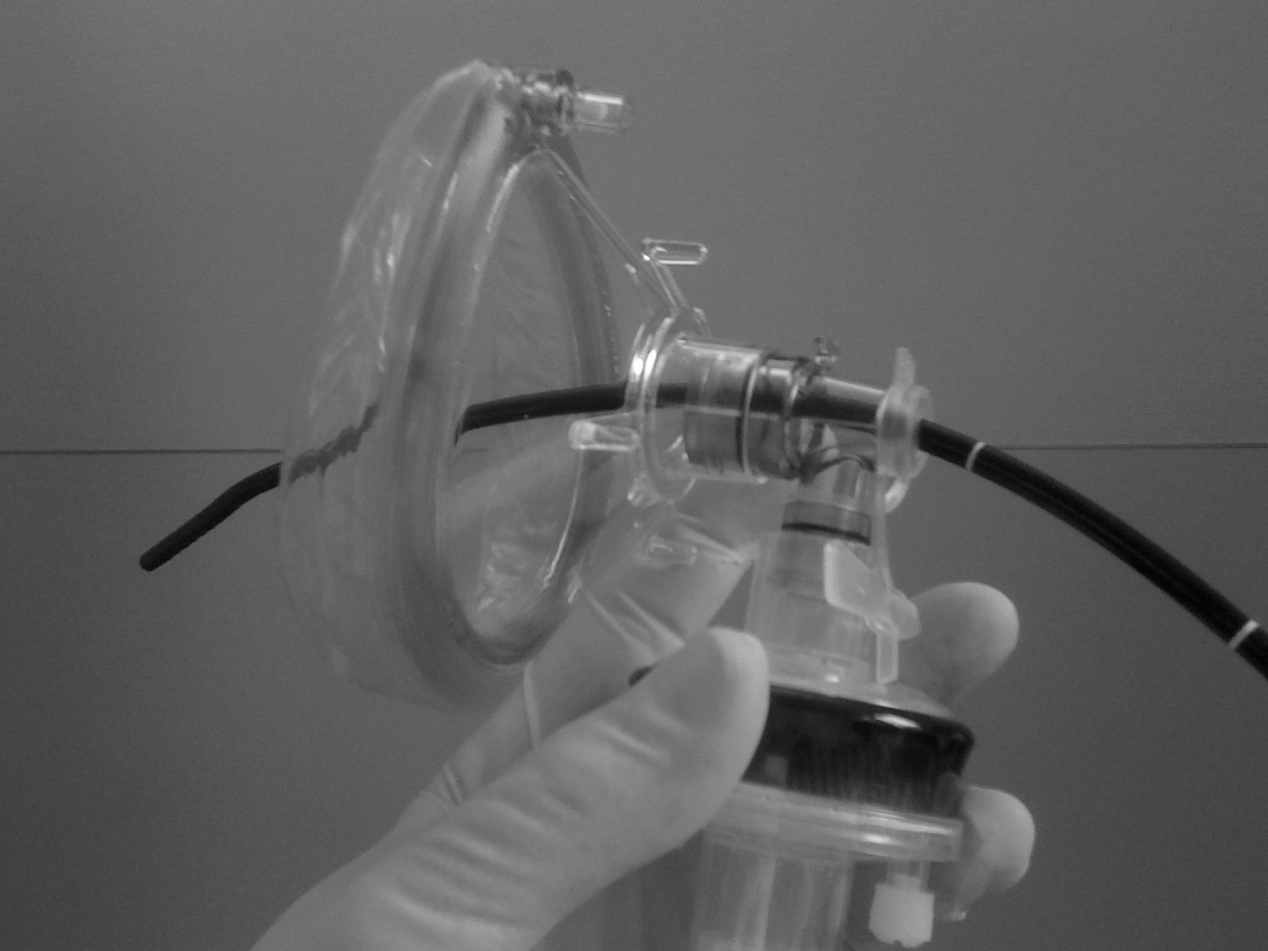
**Bronchoscope inserted through swivel connector in the face mask**.

Before, during and after the bronchoscopy, the electrocardiogram, invasive blood pressure, pulse oximeter (Infinity Omega, Dräger, Germany) and ventilatory parameters (FiO_2_, ventilator mode, in- and expiratory pressures, tidal volume, and respiratory rate) were continuously monitored. Arterial samples were drawn for blood gas analysis from the arterial line at baseline, at the end of the procedure with the bronchoscope still placed in the trachea, and 15, 30, 45, 60, and 120 minutes after removal of the bronchoscope. Ventilator settings were adjusted to optimize ventilatory support. The fraction of inspired oxygen (FiO_2_) was increased to 1.0 after the baseline blood gas analysis was obtained and kept constant throughout the procedure. After completion of bronchoscopy, FiO_2 _was reduced to the level at which it was set prior to the procedure and adjusted to maintain an arterial saturation of oxygen (SaO_2_) > 90%. Figure 1 shows the schedule of the study procedures. A swivel connector (T-adapter) was inserted between the ventilator tubing and the mask to allow the insertion of the endoscope (BF-P60, Olympus, Japan), see Figure 2. Sedation was achieved following clinical protocols using a combination of a single dose of 1 to 2 mg midazolam i.v. and repetitive bolus applications of 10 to 20 mg propofol i.v. every two to three minutes. Following application of lidocaine gel the bronchoscope was introduced nasally or orally. Topical anesthesia (5 ml lidocaine 0.8%) was applied as a spray via the bronchoscope channel to the laryngeal, tracheal and bronchial mucosa.

All bronchoscopies were performed by experienced physicians (HJB, SAB, MS, HK, SK). The decision to perform a BAL was not part of the study and was, therefore, left to the discretion of the attending intensivist. The bronchoscope was wedged in the appropriate subsegmental ostium and NaCl 0.9% solution was instilled in 20 ml aliquots and gently aspirated. The amount of BAL fluid used was determined by the number of diagnostic tests ordered. BAL fluid was subjected to microscopic analysis and culturing as previously described [16,17]. The duration of bronchoscopy was defined as the time from insertion until removal of the bronchoscope from the tracheobronchial tree.

### Outcome

Outcomes evaluated were intubation rate within eight hours of completion of bronchoscopy, intubation rate at any time, length of ICU stay, and mortality. Intubation was considered a complication possibly related to bronchoscopy, if it occurred within eight hours of the procedure. This time span was adopted from previous studies [11,18]. The decision to intubate was left to the discretion of the attending intensivists in accordance with published guidelines [14]. In addition, patients were monitored for other adverse events (for example, hemorrhage, pneumothorax, hypotension, arrhythmia) that occurred during the procedure and in the 24 hours following the bronchoscopy, which could possibly be related to the procedure.

### Statistical analysis

Results are expressed as the mean ± standard deviation (SD) for continuous variables if normally distributed and as median with 95% confidence intervals if not. For repeated measures one-way ANOVA was used to detect significant differences between cardiorespiratory parameters at baseline, at the end of the bronchoscopy, and after 15, 30, 45, 60, and 120 minutes; *P-*values of 0.05 or less were considered significant.

## Results

### Patient characteristics

The study group consisted of 40 subjects. Patient characteristics are shown in Table 1. Median PaO_2_/FiO_2 _prior to initiation of NIV was 117 ± 72, mean PaCO_2 _was 40 ± 13 mm Hg. The median duration of continuous NIV prior to bronchoscopy was 10.5 h (range 2.2 to 114 h). At baseline FiO_2 _was 0.5 ± 0.1 and was increased to 1.0 in all patients during the bronchoscopy procedure. Within 30 minutes after the bronchoscopy mean FiO_2 _level returned to the baseline level.

**Table 1:** Patient characteristics, values given as mean ± SD or no (%)

No. of patients	40
Sex ratio (m/f)	26/14
Age, years	61 ± 15
SAPS II score	47 ± 9.9
Thrombopenia < 50,000/mm^3^	6 (15)
Immunosuppression	21 (53)
Use of vasopressors	12 (30)
Antibiotic therapy	37 (93)
Antimycotic therapy	12 (30)
Antiviral therapy	5 (13)
Underlying diagnosis	
Acquired immunodeficiency syndrome	6 (15)
Hematopoietic stem cell transplantation	6 (15)
COPD	6 (15)
Thoracic surgery	4 (10)
Solid cancer	4 (10)
Hematologic malignancy	3 (8)
Pulmonary embolism	2 (5)
Pulmonary fibrosis	2 (5)
Other	7 (18)
Indication for bronchoscopy	
Suspected lower airways infection	23 (58)
Suspected retention of secretions	16 (40)
Suspected stump insufficiency	1 (3)
Ventilator mode at baseline	
Pressure support (CPAP/ASB)	30 (75)
Pressure control (BIPAP)	10 (25)

BIPAP, biphasic positive airway pressure; COPD, chronic obstructive pulmonary disease; CPAP/ASB, continuous positive airway pressure with assisted spontaneous breathing; SAPS II, Simplified acute physiology score II

Sedation was given to all patients: 27 received both midazolam (2.0 ± 0.8 mg) and propofol (69 ± 41 mg), while 13 patients (33%) received propofol alone (61 ± 33 mg).

### Tolerance of the procedure

**Figure 3:**
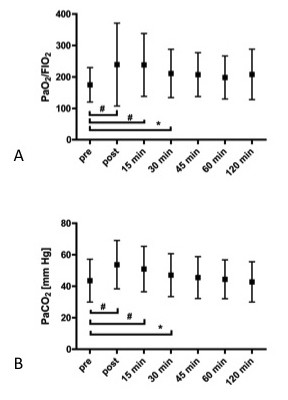
**Changes of PaO_2_/FiO_2 _(A) and PaCO_2 _(B), values given as mean ± SD**. #: *P *< 0.001 compared to baseline; *: *P *< 0.05 compared to baseline.

The average duration of bronchoscopy was 7.8 ± 5.5 minutes (range 3.5 to 37.0 minutes). In all cases bronchoscopy was completed without subsequent complications. Two patients developed transient SaO_2 _values below 90% during the procedure, the minimum SaO_2 _was 84%. Table 2 shows the course of cardiorespiratory parameters before, during and after the bronchoscopy. Significant changes from baseline were observed for PaO_2_/FiO_2_, PaCO_2 _and pH at the end of bronchoscopy, and 15 and 30 minutes after completion of the procedure, see Figure 3A. The average increase in PaCO_2 _from baseline to end of bronchoscopy was 9.4 ± 8.1 mm Hg, see Figure 3B.

**Table 2:** Cardiorespiratory parameters, values given as mean ± SD

	Baseline	End of bronchoscopy	15 minutes	30 minutes	45 minutes	60 minutes	120 minutes
Heart rate (beats/ minute)	99 ± 18	97 ± 17	97 ± 17	97 ± 17	95 ± 18	94 ± 18	92 ± 17
MAP (mm Hg)	88 ± 18	81 ± 17	83 ± 17	86 ± 17	84 ± 18	84 ± 14	83 ± 14
Respiratory rate (breaths/min)	27 ± 7	27 ± 8	30 ± 10	29 ± 9	28 ± 7	28 ± 8	27 ± 8
PaO_2_/ FiO_2_	176 ± 54	240 ± 130 *^a^*	242 ± 99 *^a^*	215 ± 76 *^c^*	207 ± 70	199 ± 68	210 ± 79
PaCO_2_ (mm Hg)	43 ± 14	53 ± 16 *^a^*	50 ± 15 *^a^*	47 ± 14 *^c^*	45 ± 13	44 ± 12	42 ± 13
pH	7.37 ± 0.09	7.30 ± 0.08 *^a^*	7.31 ± 0.07 *^a^*	7.34 ± 0.08 *^b^*	7.35 ± 0.09	7.36 ± 0.09	7.37 ± 0.08

*^a^*: *P *< 0.001 compared to baseline

*^b^*: *P *< 0.01 compared to baseline

*^c^*:*P *< 0.05 compared to baseline

MAP, mean arterial pressure; FiO_2_, fraction of inspired oxygen; PaCO_2_, partial pressure of carbon dioxide in the arterial blood; PaO_2_, partial pressure of oxygen in the arterial blood

Baseline vs. post-bronchoscopy PaO_2_/FiO_2 _did not differ between patients who underwent bronchoscopy due to suspected lower airways infection (189 ± 53 vs. 261 ± 160) vs. patients with suspected retention of secretions (159 ± 52 vs. 213 ± 92).

The amount of sedation delivered was not related to the increase in PaCO_2_. Mean heart rate and arterial pressure did not change significantly during the procedure. No patient needed initiation or dose adjustments of vasopressors during the procedure. No other adverse events, such as hemorrhage, arrhythmia, or pneumothorax, were detected.

All but two patients remained on NIV until the end of the 120-minute period following bronchoscopy. The two patients stopped NIV after 60 minutes and breathed oxygen with flow rates of 5 and 10 L/minute. Both these patients had suspected lower airways infection as the indication to perform bronchoscopy.

### Results of fiberoptic bronchoscopy

Table 3 shows the clinical and microbiological results of the bronchoscopy procedures.

**Table 3:** Results of fiberoptic bronchoscopy (*n *= 40)

No. of patients undergoing BAL	38 (95)
Instilled volume during BAL (ml)	110 (108 to 112)
Recovered BAL fluid (ml)	38 (31 to 44)
Microbiolocial results	
*Candida *species (colonisation)	18 (45)
obligate or potentially pathogenic organisms	25 (66)
*Corynebacterium*	3 (8)
*Staphylococcus aureus*	3 (8)
*Escherichia coli*	2 (5)
*Citrobacter *species	1 (3)
*Enterobacter *species	1 (3)
Methicillin resistant *Staph. aureus*	1 (3)
*Neisseria *species	1 (3)
*Proteus *species	1 (3)
*Pseudomonas *species	1 (3)
*Serratia marcescens*	1 (3)
*Pneumocystis jirovecii*	4 (11)
Cytomegalovirus	3 (8)
Herpes simplex virus	3 (8)
Bronchialcarcinoma	1 (3)
Treatment changes	13 (34)

Values given as number (%) or median (95% CI). Percent values of bronchoalveolar lavage (BAL) results refer to the group of 38 patients undergoing BAL

### Outcome

Four patients (10%) had to be intubated in the pre-specified interval of eight hours following bronchoscopy. Underlying diagnoses were suspected pneumonia in AIDS, suspected pneumonia in leukemia, and suspected retention of secretions in two patients following thoracic surgery. These events were considered to be related to the bronchoscopy. None of them were hypercapnic at baseline, but their PaO_2_/FiO_2 _ratio under NIV was significantly lower than the rest of the study population (136 vs. 180, *P *< 0.05). Two of the four patients died, one patient on the subsequent day due to cardiogenic shock, the other 25 days later. In addition, in the first 48 hours another patient died without being intubated due to progressive liver failure.

In total, intubation was necessary in 22 (55%) patients. The median interval from bronchoscopy to intubation was 27.4 h (range 2.3 to 260 h, 95% CI 23.3 to 73.8 h). Forty-eight hours after the bronchoscopy a total of 18 patients (45%) were intubated. Overall ICU mortality was 37.5% (15/40), hospital mortality 43% (17/40).

## Discussion

Our results demonstrate a low complication rate for fiberoptic bronchoscopy in critically ill patients with acute hypoxemic respiratory failure requiring noninvasive ventilation already prior to bronchoscopy. Only four patients needed endotracheal intubation during the first eight hours following bronchoscopy (10%), a result which falls within acceptable range.

The main difference of our study population to that of pre-existing studies [7-13,18,19] on bronchoscopy under noninvasive ventilatory support is that NIV had already been established in our patients irrespective of a planned bronchoscopy. In the above mentioned previous studies NIV was initiated primarily to facilitate bronchoscopy. These differences of timing and reason for NIV characterize our study population as more critically ill in terms of hypoxemia than those of previous studies. This needs to be taken into consideration when interpreting the average PaO_2_/FiO_2 _ratio of 176 ± 54 in our study, since it was determined under conditions of NIV as opposed to the average PaO_2_/FiO_2 _ratios reported in the above mentioned studies, which were recorded in spontaneously breathing patients. The mean PaO_2_/FiO_2 _of 117 ± 72 under spontaneous breathing prior to NIV initiation in our population represents one of the lowest value when compared to the existing literature.

The difference in disease severity is also reflected in a higher average SAPS II score (47 ± 9.9) of our population in comparison to that of previous studies. To our knowledge, the present study is the largest of its kind. Previous reports describe small numbers of patients under NIV requiring bronchoscopy [7,10,18,20]. However, our study, enrolling 40 patients, represents the most extensive and comprehensive report on critically ill patients requiring NIV due to acute respiratory failure and subsequent bronchoscopy.

It is difficult to determine whether the intubation of four patients (10%) within the pre-specified period of eight hours following bronchoscopy was due to the natural clinical course of the underlying disease or related to the intervention itself. In several reports studying NIV-failure rates in patients with acute hypoxemic respiratory failure it was consistently shown that 1) this patient group has *per se *an intubation rate exceeding 50% and 2) once intubation is necessary this occurs within the first 24 to 48 hours in the majority of patients [21-24].

In comparison, our patients who had been on NIV for a median duration of 10.5 hours prior to bronchoscopy required an intubation in 45% of cases within the first 48 hours following bronchoscopy. Therefore, we believe that the reported intubation rate of 10% in the first eight hours after bronchoscopy falls within an acceptable range.

Surprisingly we did not find significant changes in the gas exchange before and after bronchoscopy between the two groups of patients with suspected lower airways infection versus suspected retained secretions. This observation might be explained by the small clinical differences of these two groups: While almost all patients (38/40) underwent BAL, only very few patients showed relevant amounts of secretions in contrast to prior expectations before the procedure. The increase in PaO_2_/FiO_2 _ratio immediately after bronchoscopy cannot be explained by removal of secretions but may be attributable to other mechanisms.

Previously reported studies on bronchoscopy under NIV involved different methods of sedation. While Antonelli *et al. *exclusively used topical anaesthesia [7-9], others reported on sedation in subgroups of "agitated" patients [12]. Hilbert *et al. *described difficulties in performing bronchoscopy in severely hypoxemic patients without sedation and used analgosedation in all patients [18]. In our study, all patients received mild sedation using a combination of propofol and midazolam or propofol alone. We observed a mean increase of PaCO_2 _of 9.4 ± 8.1 mmHg that is in accordance with the results of studies on sedation during bronchoscopy in less critically ill and spontaneously breathing patients.

Our study has some limitations. First, we do not know whether performing bronchoscopy without sedation would have resulted in smaller increases of PaCO_2 _and possibly fewer intubations. As procedural sedation is the routine standard of care in our institution and no reports exist of bronchoscopy under NIV in similar patients, we considered it inappropriate to perform a controlled study.

Second, it should be emphasized that experienced clinicians performed all bronchoscopies. Therefore, our results may not be generalizable to other settings and, if less experienced physicians perform the procedures, a more cautious strategy may be appropriate. Third, as all results are influenced by the studied population, our conclusions are only applicable to patient populations with similar characteristics.

## Conclusions

We found that bronchoscopy can be performed with acceptable risk in patients with severe acute hypoxemic respiratory failure already on noninvasive ventilatory support. Since these patients *per se *have a high risk of endotracheal intubation secondary to NIV failure, bronchoscopy should only be performed by clinicians experienced with bronchoscopy and endotracheal intubation in an environment with a high level of monitoring.

## Key messages

• Noninvasive ventilation (NIV) facilitates bronchoscopy in patients with acute hypoxemic respiratory failure.

• Our study shows that even patients already requiring NIV due to acute hypoxemic respiratory failure may undergo bronchoscopy with acceptable risk.

• As these patients *a priori *have a high risk of NIV failure, only experienced staff should perform bronchoscopy in this patient population.

• In our study, BAL results revealed obligate or potentially pathogenic organisms in 25/38 patients (66%) and lead to treatment changes in 13/38 (34%) of patients.

## Abbreviations

BAL: bronchoalveolar lavage; BIPAP: biphasic positive airway pressure; CPAP/ASB: continuous positive airway pressure with assisted spontaneous breathing; ICU: intensive care unit; NIV: noninvasive ventilation; PaO_2_/FiO_2_: ratio of partial pressure for oxygen to fraction of inspired oxygen; PEEP: positive end-expiratory pressure; SaO_2_: arterial oxygen saturation; SAPS II: Simplified Acute Physiology Score II; SD: standard deviation

## Competing interests

The authors declare that they have no competing interests.

## Authors' contributions

HJB, HK, MS, SB, JKH and SK have made substantial contributions to the conception and design of the study. HJB, HK, MS, SAB and SK performed the bronchoscopies. TG collected the data. HJB, TG and SK performed the analysis and interpretation of data. HJB, SAB and SK drafted the manuscript. All authors read and approved the final manuscript.
